# Luminal Surface Plasma Treatment of Closed Cylindrical Microchannels: A Tool toward the Creation of On-Chip Vascular Endothelium

**DOI:** 10.1021/acsbiomaterials.2c00887

**Published:** 2023-04-27

**Authors:** Marek Černík, Kamila Poláková, Lukáš Kubala, Andrea Vítečková Wünschová, Anna Mac Gillavry Danylevska, Michaela Pešková, Jan Víteček

**Affiliations:** † Institute of Biophysics of the Czech Academy of Sciences, Královopolská 135, 612 65 Brno, Czech Republic; ‡ Department of Biochemistry, Faculty of Science, Masaryk University Brno, Kamenice 5, 625 00 Brno, Czech Republic; § International Clinical Research Center, St. Anne’s University Hospital Brno, Pekařská 53, 656 91 Brno, Czech Republic; ∥ Department of Anatomy, Faculty of Medicine, Masaryk University Brno, Kamenice 5, 625 00 Brno, Czech Republic; ⊥ Department of Histology and Embryology, Faculty of Medicine, Masaryk University Brno, Kamenice 5, 625 00 Brno, Czech Republic

**Keywords:** 3D printing, endothelial cell, in vitro model, plasma oxidation, PDMS, surface modification

## Abstract

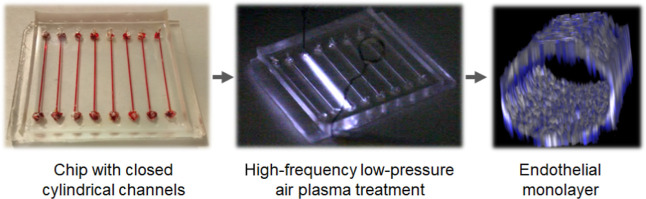

On-chip vascular microfluidic models provide a great tool to study aspects of cardiovascular diseases in vitro. To produce such models, polydimethylsiloxane (PDMS) has been the most widely used material. For biological applications, its hydrophobic surface has to be modified. The major approach has been plasma-based surface oxidation, which has been very challenging in the case of channels enclosed within a microfluidic chip. The preparation of the chip combined a 3D-printed mold with soft lithography and commonly available materials. We have introduced the high-frequency low-pressure air-plasma surface modification of seamless channels enclosed within a PDMS microfluidic chip. The plasma treatment modified the luminal surface more uniformly than in previous works. Such a setup enabled a higher degree of design freedom and a possibility of rapid prototyping. Further, plasma treatment in combination with collagen IV coating created a biomimetic surface for efficient adhesion of vascular endothelial cells as well as promoted long-term cell culture stability under flow. The cells within the channels were highly viable and showed physiological behavior, confirming the benefit of the presented surface modification.

## Introduction

Polydimethylsiloxane (PDMS) is the most widely used material to produce microfluidic chips due to its transparency, gas permeability, and no toxicity for cell cultures. It is, however, highly hydrophobic.^1^ For biological applications, suitable wetting properties are a prerequisite for good cell adhesion and stable cell culture. These are generally achieved by making the surface hydrophilic using plasma oxidation,^2−4^ silanization,^5,6^ polydopamine,^7^ or multiple-step surface modifications.^8^ However, additional molecular patterns that can be recognized by cell adhesion receptors are required as well.^9^ Hence, the surface of a material can be further functionalized by the deposition of proteins from the extracellular matrix that naturally contain such molecular patterns, for example, fibronectin,^10^ matrigel,^11^ collagen,^12^ or gelatine.^13^

A variety of on-chip vasculature models have been established. The commercially available microfluidic chips (e.g., IBIDI or Cellix) are excellent in terms of surface chemistry; however, they mostly disregard the circular cross-section of the channel, which is essential for the proper function of endothelial cells.^14,15^ Although these devices provided insights into vascular biology, precise studies may fail due to biological consequences of unwanted flow disturbances resulting from channel geometry, for example, edges. It can cause nonphysiological behavior, which can be observed as a change in cell morphology.^10,16^ The disturbance of shear stress is an initial point of development of many cardiovascular diseases.^16^ That is why an on-chip vascular model having a circular cross-section of channels is beneficial to the field.^16−18^

A set of procedures has been introduced to create channels with circular cross-sections in a PDMS-based chip. A basic approach is to create a rectangular channel that is further modified into a cylindrical one by additional material deposition.^19,20^ Cylindrical channels have been often created by assembling two complementary PDMS casts. Huang and his colleagues used a photoresist reflow to prepare a mold for semi-cylindrical channels. The PDMS casts were plasma-treated and bonded under an optical microscope to render cylindrical channels.^14^ Vecchione used spin coating of PDMS onto the surface of a rectangular channel prior to PDMS cast bonding.^21^ Another set of approaches used a removable cylindrical molding element, dissolvable^22^ or permanent,^10,23^ embedded in a chip cast, leaving an open channel after removal.^10,22,24^

Alternatively, 3D printing could be used for the preparation of microfluidic devices. It enables rapid prototyping with a high degree of uniformity and reproducibility. The limitation is a printer resolution which affects the minimum reasonable channel diameter. Furthermore, the resolution strongly influences the channel lumen roughness.^24−26^ Therefore, it could be convenient to combine the benefits of 3D printing with the precision of molding elements.

Plasma treatment in a closed microfluidic channel is very challenging. The standard procedure of plasma-based surface modification is carried out in a plasma chamber.^3^ However, it is not readily applicable to long and narrow channels enclosed in a chip.^4^ A well-controlled surface modification would require the discharge inside a closed channel. Indeed, such a procedure was implemented using air^2^ or helium^4^ at ambient pressure,^2^ but it may result in non-uniform surface modification.^2^ The uniformity of the surface plasma modification could be improved by lowering the pressure and using a high-frequency discharge.^27^

In this paper, we introduced high-frequency low-pressure air-plasma-based surface modification of seamless channels enclosed within a PDMS microfluidic chip in order to promote a stable culture of vascular endothelial cells under flow. Such a surface modification is an integral part of a more complex procedure, which allows for rapid prototyping of microfluidic chips. This model could be used to study aspects of cardiovascular diseases in vitro.

## Materials and Methods

### Mold Design and Preparation

**Figure 1:**
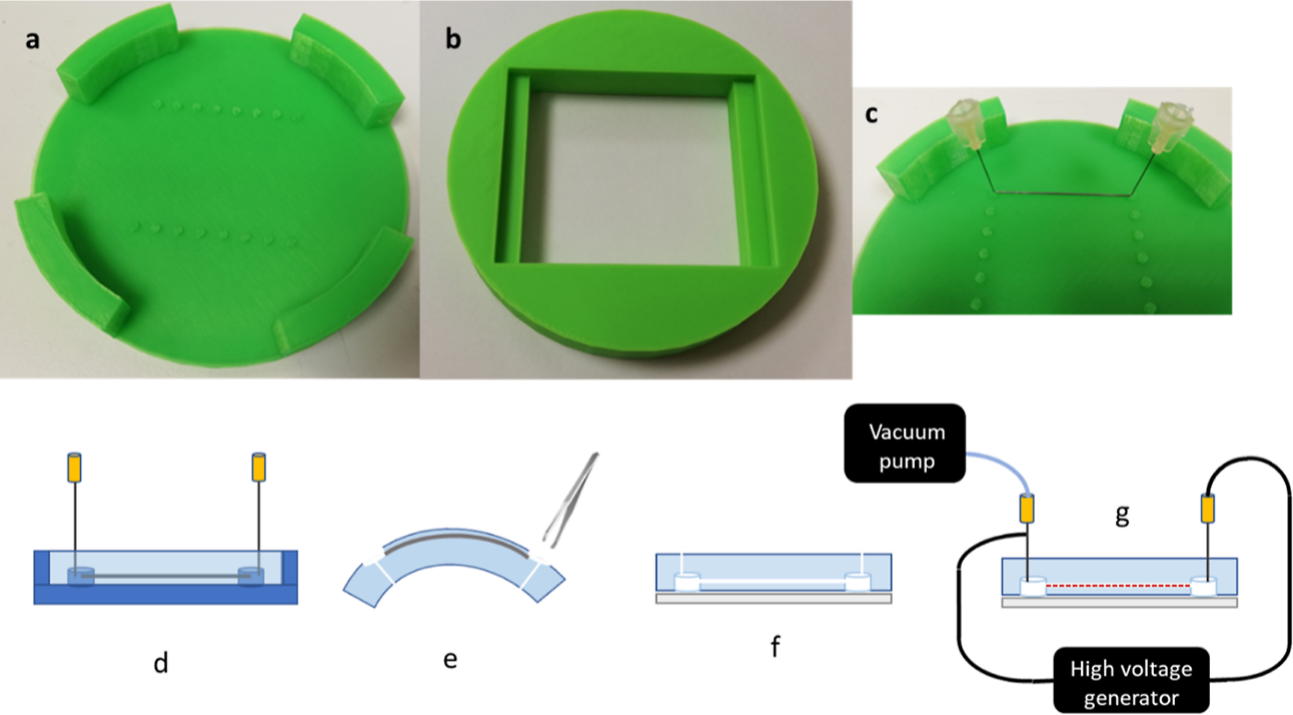
3D-printed mold for chip casting (a–c) and scheme of preparation of a microfluidic chip (d–g). (a) Unsmoothed 3D-printed mold for the microfluidic chip (bottom part). The mold was printed using acrylonitrile-butadiene-styrene. (b) The upper part of the 3D-printed mold. (c) Detailed image of the alignment structure holding molding elements. The alignment structures on the mold base are the key features that served as a scaffold for the molding elements for future channels and interfaces. (d) Scheme of mold assembly (dark blue). Molding elements were coated with BSA to limit adhesion to PDMS. They were placed into alignment structures, and liquid PDMS was poured inside the mold and cured. (e) Molding elements removal. After curing molding elements for inlets and outlets (stainless steel needles) were removed while the PDMS bulk was still inside the mold. Cured chip bulk was removed from the mold and bent to create a wide opening in the imprint of the alignment structure so that the molding elements for channels (stainless steel entomological pins) could be removed. (f) Sealing of the microfluidic chip. PDMS bulk was plasma-bonded to glass to seal the holes left by alignment structures. (g) Plasma treatment of the channel’s luminal surface. Channels in the chip were subjected to high-frequency low-pressure air plasma treatment using a portable high-voltage generator.

The mold was designed in AutoCAD software (Autodesk, USA, stl files available in the Supporting Information) and 3D-printed (Prusa i3 MK2, Prusa Research, Czech Republic, nozzle 250 μm, resolution 150 μm) from acrylonitrile butadiene styrene (ABS, Gembird Europe, The Netherlands, see Figure 1 panels a–c). The mold featured alignment structures for precise alignment of molding elements that were covered with a thin layer of 70% polyethylene glycol (PEG, CarboWax, Serva, Germany) water solution. The printed mold was then smoothened using an acetone vapor chamber (briefly: the inner surface of a 1 liter beaker was covered with acetone-soaked paper towel, and the beaker was tightly closed. The printout was quickly inserted onto an inverted Petri dish at the bottom 5 min after vapor saturation. The mold was treated at room temperature for 40 min. The effect of such treatment on mold roughness is demonstrated in Figure S1). Residual PEG was carefully rinsed with distilled water.

### Chip Preparation

Stainless steel entomological pins (cat. no. 02.01, Ento Sphinx, Czech Republic) with 400 μm diameter (variability among pins within ±20 μm, longitudinal variability in one pin ±5 μm, smooth surface) were used as molding elements to create channels. The interface was molded using standard 23 G syringe needles (NN-2332R, Terumo, Belgium) with a 300 μm diameter. All molding elements were pre-coated with a 1% solution of bovine serum albumin (BSA, Merck) in distilled water for 5 min at RT. Molding elements were washed by immersion into distilled water and blown dry with air. After drying, they were placed into alignment structures. Liquid degassed PDMS (Sylgard 184, Dow Corning, USA) was poured into the mold (Figure 1d). PDMS was mixed in a standard 10:1 ratio of base and curing agent. After curing at 70 °C for 2 h, the PDMS was let to cool down. Molding elements for the interface (stainless steel needles) were removed while the PDMS bulk was still inside the mold. The cured chip bulk was then peeled off the mold, and the molding elements from channels (stainless steel entomological pins) were gently removed (Figure 1e). The bottom of the chip was then sealed (Figure 1f) with a custom-cut glass (39 × 49 mm, glass no. 1, Menzel-Gläser, Germany) by plasma-assisted bonding.^28^

### Channel Slice Preparation

Channels surrounded with a bit of PDMS bulk were cut out of a microfluidic chip using a surgical blade. These were then cut into smaller segments that were embedded into CryoMount medium (Histolab, Sweden) and left at −20 °C overnight. The following day, the segments were cut into 20 μm thick slices perpendicular to the channel using Cryotom (Leica CM 1800, Germany). The slices were then observed using an AxioObserver Z1 microscope (Zeiss, Germany).

### Surface Modification of PDMS

Prior to application into the chip channels, all surface modifications were tested using PDMS discs (1 mm thick, 15 mm in diameter). The Piranha solution was prepared as a mixture of sulfuric acid and 30% (w/w) hydrogen peroxide in a 3:1 ratio. The procedure was adopted from Koh et al.^29^ 200 μL of solution were placed on each disc’s surface, and the discs were incubated in an open Petri dish at 50 °C for 30 min. After the modification, the discs were thoroughly rinsed with distilled water and dried.

Both untreated and Piranha solution-treated discs were used for silanization in a solution of tetraethyl orthosilicate (TEOS, Merck, cat. no. 86578), ethanol, and 0.1 M HCl in a 1:3:1 ratio. This mixture was preincubated in a tube with an inert atmosphere for 18 h at 37 °C, according to Abate et al.^30^ 200 μL of solution were then placed on the preheated disc’s surface, and the discs were incubated in a closed Petri dish at 90 °C for 5 min. After the modification, the discs were thoroughly rinsed with distilled water and dried.

The high-frequency low-pressure air plasma treatment (further referred to as plasma treatment) of the discs was done using corona discharge generated by PEP-12 electromassage apparatus (Elfa-Srb, Blatná, Czech Republic). PDMS discs were placed on a conductive substrate (aluminum foil) to which a grounding electrode was connected. The working electrode was placed 2 mm above the PDMS surface. The PDMS discs were treated with plasma for 1 min each.

The untreated as well as the plasma-treated discs were immediately sterilized with 70% ethanol for 5 min. Next, the discs were washed with sterile water and modified with collagen types I and IV. For collagen type I, a solution of collagen in phosphate-buffered saline (PBS, composition: 1 liter of distilled water with the addition of 0.2 g KH_2_PO_4_; 8 g NaCl; 2.3 g Na_2_HPO_4_; and 0.2 g KCl; pH 7.4) was used at a final concentration of 0.1 mg mL^–1^. For collagen type IV, a solution of collagen in PBS with a 5-fold higher concentration of phosphate was used at the final concentration of 0.13 mg mL^–1^. The discs with collagen solutions were incubated for 1 h at 37 °C. Subsequently, collagen solutions were removed from the discs, and the discs were rinsed with PBS.

### Contact Angle Measurement

A 5 μL drop of distilled water was applied to the discs to be examined. The drop was photographed with a Dino-Lite CCD camera using DinoCapture software 2.0 (AnMo Electronics Corporation, Taiwan). The image analysis was carried out in ImageJ^31^ using the manual procedure in the Contact Angle plugin.

### Cell Culture

Murine endothelial MS1 cells (Mile Sven 1, ATCC, cat. no. CLR-2279) were cultured on plastic Petri dishes (culture treated, TPP, Switzerland, cat. no. 93100) in DMEM medium (Gibco, cat. no. 11995065) with the addition of 10% fetal bovine serum (cat. no. P30-3302, PAN Biotech) and 1% of streptomycin and penicillin (cat. no. P06-07050, PAN Biotech). Cultivation was carried out at 37 °C. The air in the incubator contained 5% CO_2_ and had 100% humidity.

### Biocompatibility Evaluation of Surface Modifications

The effectivity of the modifications was determined using a cell culture viability assay. Sterilized (see above) PDMS discs were placed into a 24-well cell culture plate and modified with collagens where required. Empty wells served as controls. A suspension of mouse endothelial cells (MS1 line) in culture medium (DMEM, Gibco) was then added to the wells at 20,000 cells per well. The cells were cultured for 2 days. The viability of cell culture was evaluated based on the level of intracellular adenosine triphosphate (ATP)^32^ using an ATP assay kit (BioVision, K355-100). The culture medium was removed, and cells were rinsed with ice-cold PBS. It was followed by lysis in 250 μL of cold lysis buffer on a shaker at 4 °C for 15 min. Lysates were clarified by centrifugation (5000*g*, 4 °C, 5 min). The rest of the procedure was carried out according to the instructions of the kit manufacturer.

### Plasma Modification of Luminal Chip Surfaces

The chip was modified using PEP 12-generated plasma. A vacuum (15 kPa of residual pressure) was introduced into the chip inlet through the needle. The needle also served as an electrode. The second electrode consisted of a steel wire that was inserted into the outlet opening at the other end of the channel (Figure 1g). The luminal surface of the channel was treated with plasma discharge for 1 min.

### Plasma Uniformity Determination

Channels of a freshly prepared microfluidic chip were modified with plasma for 1 min (for details, see the previous chapter). Channels were subsequently coated with rhodamine b-labeled BSA solution (0.87 mg mL^–1^), prepared as described in Nikitin et al.,^33^ for 1 h at 37 °C. After coating, channels were thoroughly washed with PBS and were imaged under a microscope (AxioObserver Z1, Zeiss, Germany) in 1 mm sections. Due to unpredictable light scattering close to the ends of channels (which was related to the edge of channel inlet/outlet structures), a limited length of channels was assayed (22 mm out of the 30 mm channel). The overall fluorescence intensity was determined using the ImageJ^31^ after subtracting the background (rolling ball, 50 px).

### Electron Microscopy

Collagen IV-coated channels were fixed with 3% glutaraldehyde in a 0.1 M cacodylate buffer. The samples were washed three times with a 0.1 M cacodylate buffer. All three sample types: native PDMS, plasma-treated PDMS, and collagen-coated samples were embedded into Cryomount medium (Histolab, Sweden) and left at −20 °C overnight. Approximately one-half of the channel was cut off longitudinally using Cryotom (Leica CM 1800, Germany) to expose channel lumen. The residual Cryomount medium was washed with 0.1 M cacodylate buffer. Samples were dehydrated using an ascending ethanol grade and dried using the critical point method in a CPD 030 dryer (BAL-TEC Inc., Liechtenstein), coated with gold using a sputter coater (SCD 040, Balzers Union Limited, Liechtenstein) and examined on a scanning electron microscope (VEGA TS 5136 XM, Tescan Orsay Holding, Czech Republic) using the secondary emission detector and 20 kV acceleration voltage.

### Cell Seeding and Cultivation under Flow

A microfluidic chip μ-Slide I Luer (cat. no. 80176, IBIDI Gräfelfing, Germany), connected to the IBIDI pump system (cat. no. 10902, IBIDI Gräfelfing, Germany), was used as a reference microfluidic chip. Briefly, 150 μL of suspension of MS1 cells at 1.2 × 10^6^ to 2.5 × 10^6^ cells per mL was loaded into a chip, and the cells were left to adhere for 2 h. The chip was connected to the fluidic unit with a total of 12 mL of culture medium to recirculate through the chip in a unidirectional manner. Initial perfusion was set to achieve a shear stress of 0.2 Pa. The shear stress was gradually incremented in 2 h intervals to reach 0.5, 0.7, 1, and finally 1.5 Pa (an optimum for MS1 cells on a microfluidic chip μ-Slide I) under which the cells were kept for 5 days under a 5% CO_2_ atmosphere. Medium exchange in reservoirs was carried out in 2 days intervals.

Channels in our chip were seeded with a suspension of murine endothelial MS1 cells in a cultivation medium (10 μL per channel, density 10^6^ cells per mL). 5 min after the seeding, the chip was gradually rotated in 90° increments (four times, 5 min each) to ensure uniform cell coverage all over the luminal surface of the channels. The cells were left to adhere for 2 h, and the chip was connected to a microfluidic pump system. Either the IBIDI pump system (see above) or the Kima pump (Cellix, Dublin, Ireland) was used to cultivate the cells under constant unidirectional flow with shear stress of 0.03 Pa (an optimum for MS1 cells on this microfluidic chip; the cell response to flow in terms of morphology did not change for shear stress 0.03 Pa up to about 5 Pa when wash off started) under 5% CO_2_ atmosphere for 5 days. In the case of the IBIDI system, the fluidic unit reservoirs contained 12 mL of culture medium, which was exchanged in 2 day intervals. For the Kima pump, 50 ml of culture medium was recirculated for the whole duration of the experiment.

### Cell Viability Assay

The cell viability for both models was checked using vital staining with 2 μM fluorescein diacetate (FDA) and 20 μM propidium iodide (PI) according to Vitecek et al., 2007^34^ using a fluorescent microscope (AxioObserver Z1, Zeiss, Germany). Green fluorescent cells stained with FDA were considered alive, whereas red fluorescent cells stained with PI were considered dead.

### Cell Staining and Microscopy

Cells within the channels were fixed with 3% formaldehyde, followed by permeabilization using 0.1% Triton X-100 in PBS and blocking for 1 h at RT in 5% BSA (Sigma-Aldrich, USA) in PBS. F-actin was visualized using phalloidin-conjugated Alexa Fluor 488 (3 U/mL; Invitrogen, Life Technologies, USA) for 1 h at RT. In another experiment, zonula occludens 1 (ZO1) was visualized using anti-ZO1 antibody (1:100, cat. no. 33-9100, Thermo Fisher Scientific, USA) combined with Alexa Fluor 488-labeled secondary antibody (1:150, cat. no. A-11001, Thermo Fisher Scientific, USA). Nuclei were stained with DAPI (0.4 μg/mL). After the staining, channels were filled with PBS, and the images were acquired a fluorescent microscope (AxioObserver Z1, Zeiss, Germany). For 3D reconstruction imaging with F-actin and nuclei staining, a confocal microscope (TSC SP-5 X, Leica, Germany) equipped with N Plan 10×/0.25 PH1 objective and LAS AF software (Leica, Germany) was utilized. 3D reconstruction was rendered using the 3D viewer plugin in ImageJ.^31^

### Image Analysis of Cell Elongation

The cell elongation in the direction of flow was assessed by image analysis of photographs obtained throughout the cultivation. All the image analyses were performed in ImageJ software.^31^ At least 300 cells out of two biological replicates were processed. The elongation index was calculated as a ratio of the major and minor axes of the circumscribed ellipse to individual cells.

## Results

3D-printed mold (see Figure 1a–c, stl files available in the Supporting Information) based on ABS copolymer was used to produce a PDMS microfluidic chip. Surfaces to be in contact with PDMS were polished using the acetone vapor approach (Figure S1). The mold had alignment structures, which served as a scaffold for molding elements for channels and the interface. To avoid etching of these structures, PEG masking was applied.

**Figure 2:**
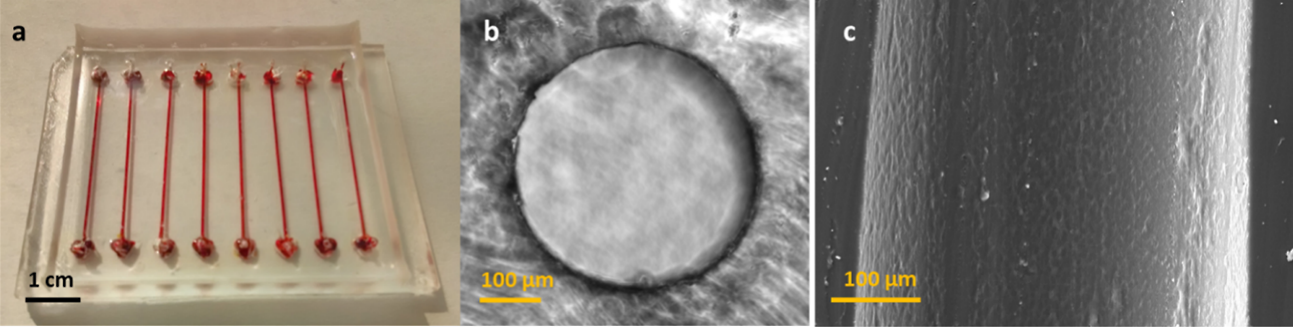
(a) Photo of a microfluidic chip (length 49 mm, width 39 mm). The chip consists of 8 linear channels with inlets and outlets. Channels are 3 cm long with a diameter of 400 μm. (b) Brightfield image of empty channel lumen in native PDMS. The channel segments were sectioned into 20 μm slices and observed under a microscope. A typical image is displayed. (c) SEM image of the channel’s luminal surface in native PDMS. A typical image is displayed.

The molding elements (stainless steel pins) were used to create cylindrical channels within the PDMS chip (Figure 2a). The diameter variability of a single channel was within 5 μm. Without any surface modification, molding elements removal from cured PDMS resulted in severe damage to the luminal surface of channels (not shown). To avoid such issue, the molding elements were coated with BSA. The modification allowed their easy removal without major scratches, as seen in the channel cross-section examined with light microscopy (Figure 2b) and in the longitudinal section examined with electron microscopy (Figure 2c).

**Figure 3:**
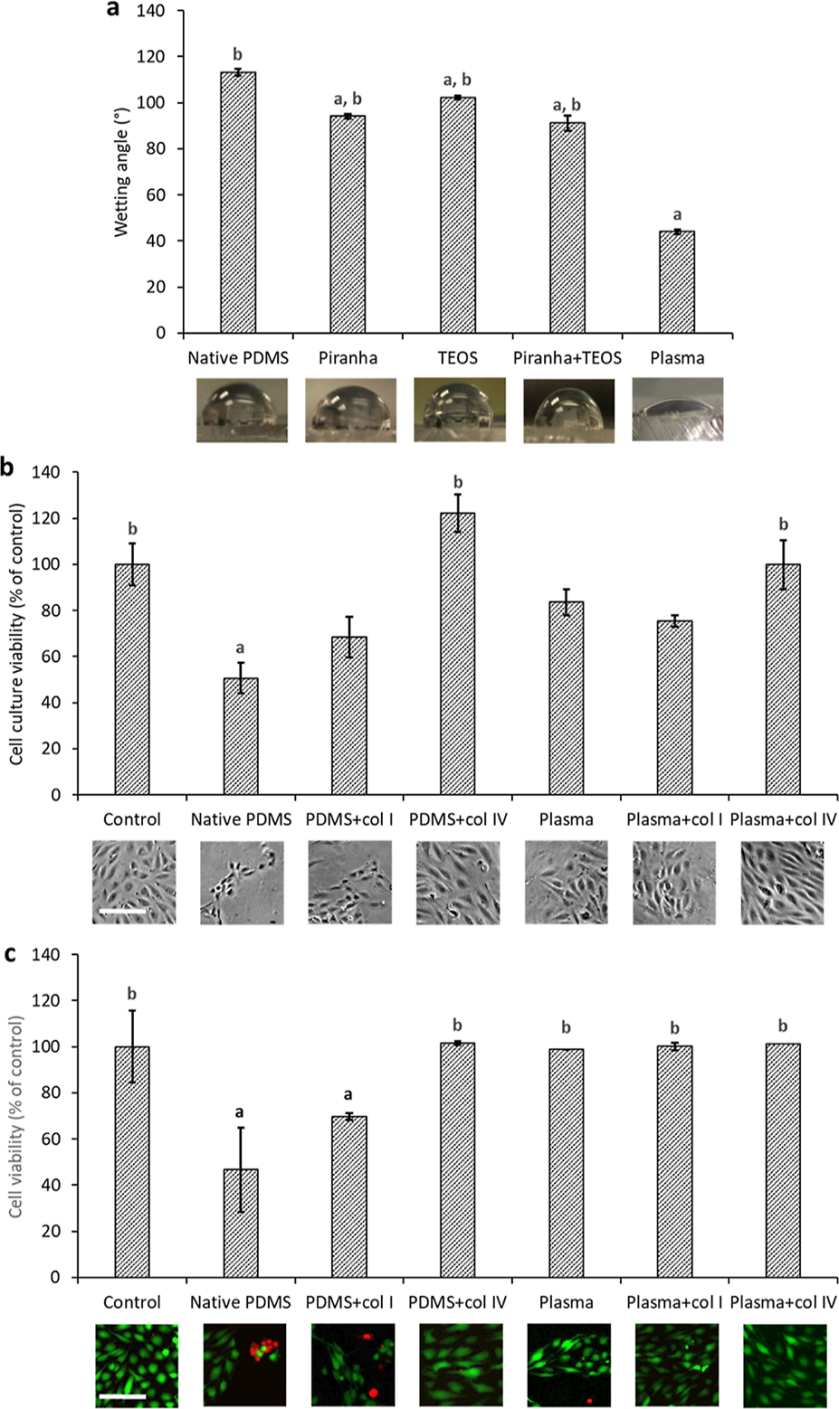
(a) Comparison of the effects of Piranha solution oxidation, silanization (tetraethyl orthosilicate—TEOS), their combination, and plasma oxidation on lowering the wetting angle of native PDMS. *N* = 6, data shown as mean ± SEM. Dataset was analyzed using one-way ANOVA with the Tukey post hoc test (*p* 0.05). Symbols a and b stand for statistical significance compared to native PDMS and to plasma oxidation, respectively. For a complete table of *p*-values, see Supporting Information (Table S1). Images of water droplets on the respective substrate are displayed below the chart. (b,c) Comparison of the suitability of plasma surface oxidation, protein coating, and their combinations for the culture of MS1 endothelial cells. The cell culture viability was determined by intracellular ATP assay (b), whereas the cell viability was quantified as the ratio of living cells by image analysis of vital staining with fluorescein diacetate and propidium iodide (c). Data are shown as a percentage of control—cell culture plastic (100%) cells that were cultured for 2 days; control—standard cell culture plastic, PDMS—native PDMS, PDMS + col I—native PDMS coated with collagen I, PDMS + col IV—native PDMS coated with collagen IV, plasma—native PDMS oxidized by plasma, plasma + col I—native PDMS oxidized by plasma and coated with collagen I afterward, plasma + col IV—native PDMS oxidized by plasma and coated with collagen IV afterward; data shown as mean ± SEM (*N* = 3–6). Dataset was analyzed using one-way ANOVA with the Tukey post hoc test (*p* 0.05). Symbols a and b stand for statistical significance compared to control and to native PDMS, respectively. For a complete table of *p*-values, see Supporting Information (Tables S2 and S3). Representative images in phase contrast (b) and fluorescence (c; green, fluorescein-stained living cells; red, propidium iodide-stained dead cells) are displayed. Bar indicates 100 μm.

To overcome the limiting hydrophobicity of PDMS for cell culture, several surface modifications were evaluated (see Figure 3a). Prior to application to closed channels in PDMS chips, PDMS discs were used as a simplified model to select the best procedure. First of all, we compared the effect of liquid-based procedures on lowering the water contact angle of native PDMS (113.2 ± 1.0°), such as oxidation by Piranha solution (94.1 ± 1.5°), silanization (102.3 ± 1.0°) using tetraethyl orthosilicate (TEOS), and their combination (91.1 ± 0.8°) with low-pressure high-frequency air plasma oxidation (44.0 ± 3.2°, further, referred to as plasma treatment). The most effective surface modification was the plasma treatment (see Figure 3a).

To further enhance the surface biocompatibility of PDMS, we tested the collagen coating using PDMS discs. The biocompatibility was evaluated in terms of cell culture viability, cell morphology, and coverage with cells. The standard cell culture plastic was used as a reference (100%, see Figure 3b). Cell culture grown on native PDMS showed moderate viability (50.6 ± 9.1%). The collagen I coating resulted in a small added value (cell culture viability 68.5 ± 6.5%). However, collagen IV rendered a remarkable increase in cell culture viability (122.2 ± 8.8%). The plasma treatment alone had a positive effect (cell culture viability 83.6 ± 8.1%). Combined coating with collagen I, again, had no added value (75.4 ± 5.6%), whereas the additional coating with collagen IV resulted in cell culture viability at the level of standard cell culture plastics (99.8 ± 2.5%). Cell morphology and coverage with cells reached the level of control in collagen IV-coated native and plasma-treated PDMS (Figure 3b).

To examine the viability on the level of single cells, we performed vital staining using fluorescein diacetate (FDA) and propidium iodide. Compared to control (cell culture plastic), the cell viability was reduced to about 50% on native PDMS and to about 70% on native PDMS with collagen I coating. Cells growing on other substrates showed viability on the level of control (Figure 3c).

Even though the coating of native PDMS with collagen IV provided the highest viability of cell culture, additional plasma treatment before collagen IV coating was necessary for long-term cell culture stability, highlighting the importance of a hydrophilic surface for the coating. Therefore, this combination was used for further experiments on a chip.

**Figure 4:**
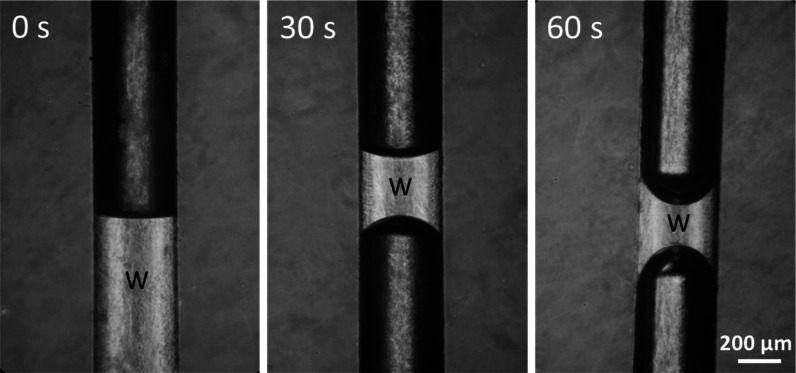
Impact of air plasma treatment on the hydrophilicity of the channel lumen. High-frequency discharge was applied to an evacuated channel. Hydrophilicity was estimated by measuring the contact angle of the water meniscus using a contact angle plugin in ImageJ software. The brighter part of the channel was occupied by water (w).

The difficult plasma treatment of the channel luminal surface was implemented by discharge in an evacuated channel. The wetting angle gradually decreased with plasma treatment time. After 60 s, a value of 23.0 ± 2.7° was achieved (Figure 4). Such treatment was applied right before the collagen IV coating.

**Figure 5:**
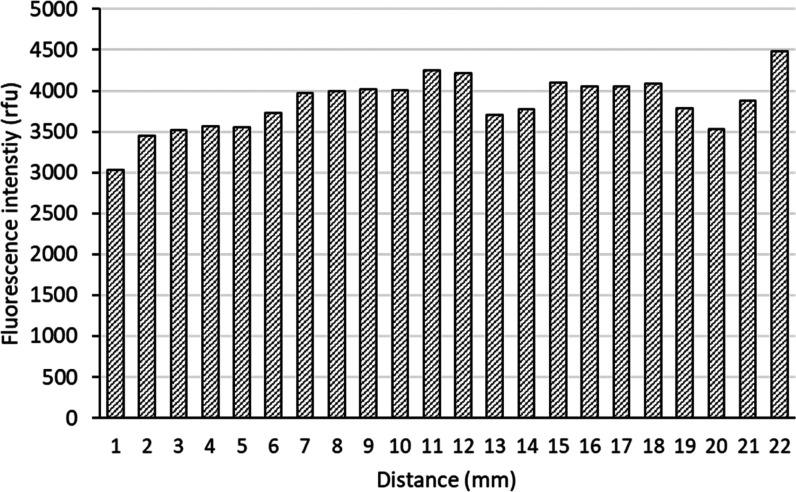
Determination of plasma treatment uniformity by means of protein adsorption. Fluorescence intensity of rhodamine b-labeled BSA adsorbed on the wall of the channel was imaged using a fluorescence microscope in 1 mm sections of a single channel. Representative dataset out of four independent replicates is displayed.

The uniformity of the plasma treatment was assayed by the adsorption of rhodamine b-labeled BSA (Figure 5). The irregularity determined as a relative standard deviation of the mean value was within ±10% for all assays of individual channels. Another assay to characterize the uniformity of plasma treatment by means of sampling for wetting angle at multiple points was charged with high experimental error (Figure S2).

**Figure 6:**
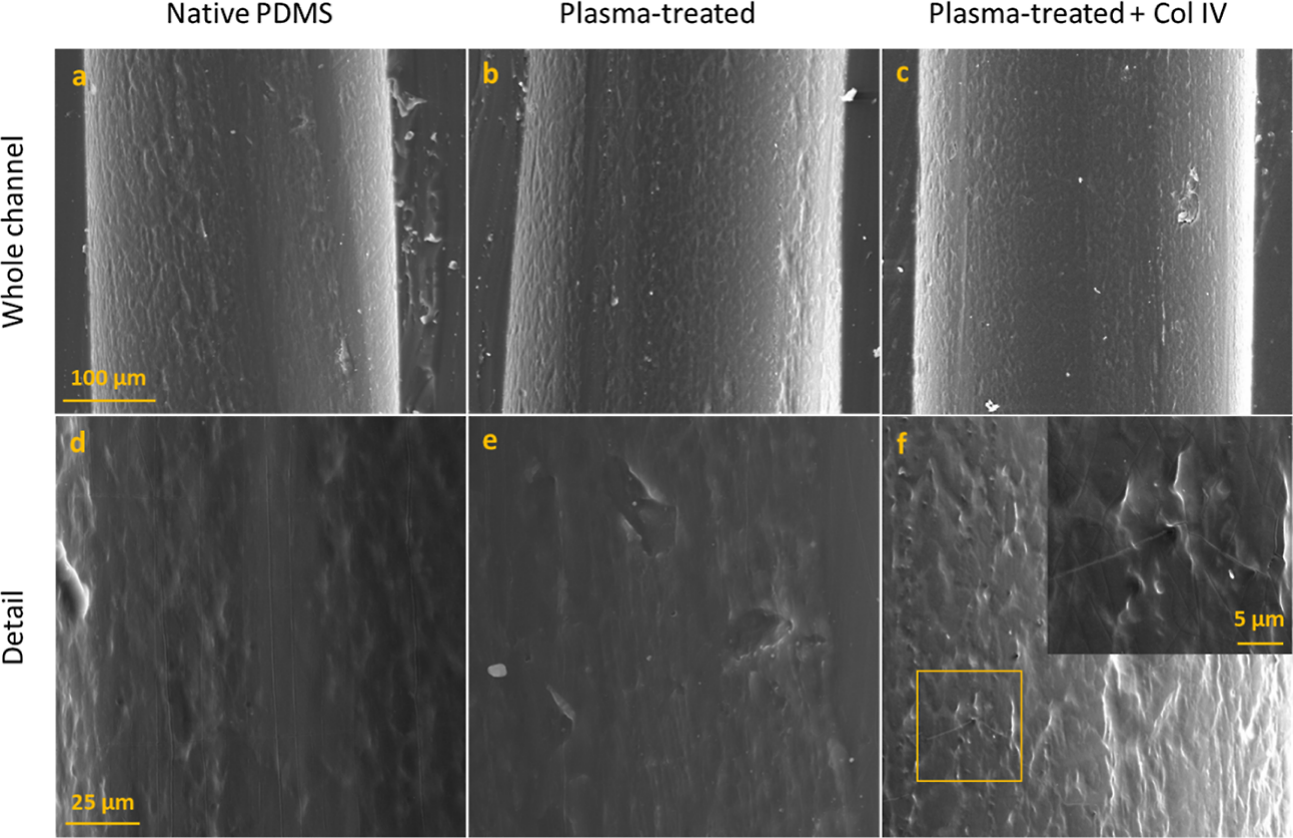
Investigation of luminal surfaces of channels by SEM. Channel sections were cut longitudinally, processed, and visualized at different magnifications. The surfaces of native PDMS (a,d), plasma-treated PDMS (b,e), and plasma-treated PDMS additionally coated with collagen IV (c,f) are displayed. In section F, the inset shows the magnified cut-out indicated by a square.

Longitudinal sections of the channels were subjected to SEM, in order to further characterize the luminal surface of the native PDMS (see Figure 6a,d), PDMS after plasma treatment (see Figure 6b,e) and plasma-treated PDMS which was additionally coated with collagen IV (see Figure 6c,f). There were visible scales on the surface of the native PDMS, which measured approximately 5 μm. Longitudinal and perpendicular imprints with a submicrometer width were observable as well. Plasma treatment did not produce major changes on the surface of the PDMS. By contrast, the PDMS coated with collagen IV displayed a wrinkled layer of collagen IV with occasional cracks on its surface (Figure 6f). Both wrinkles and cracks are likely the result of sample processing.

Additionally, the amount of collagen IV adsorbed to the channel luminal surface was estimated. The result indicated about 1.5 times higher amount of collagen bound to native PDMS compared to plasma-treated PDMS (Figure S3).

**Figure 7:**
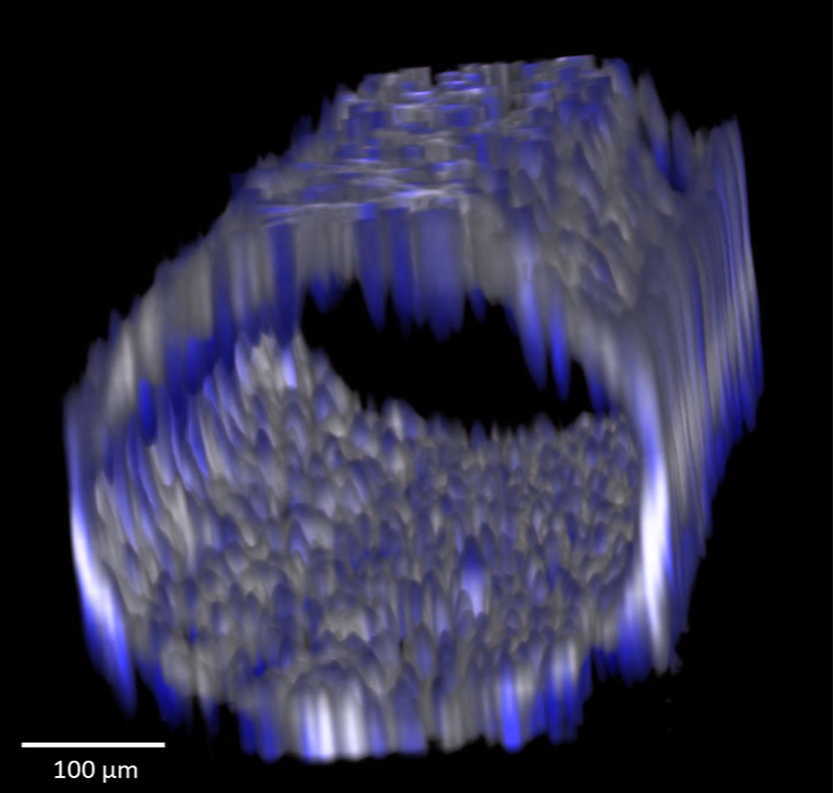
3D view of a z-stack from fluorescent confocal microscopy images of a confluent monolayer of endothelial cells inside the chip channel. A cell monolayer was accomplished after 5 days of cultivation under flow (shear stress of 0.03 Pa). Cells were stained for actin with Alexa 488-conjugated phalloidin (gray) and for nuclei with DAPI (blue).

**Figure 8:**
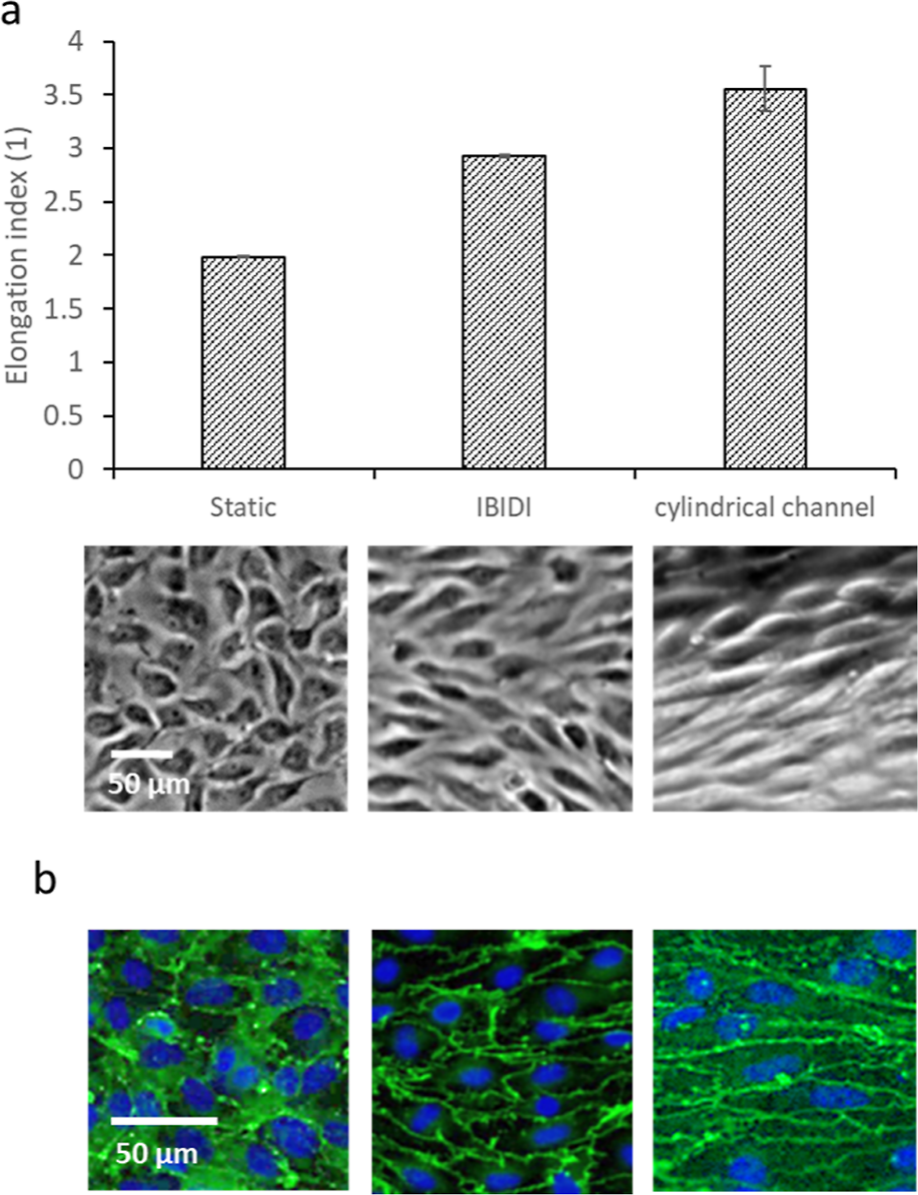
Behavior of mouse endothelial cells inside a cylindrical channel under flow after 5 days compared to a commercially available microfluidic chip (IBIDI) and static culture in terms of cell (a) elongation and (b) expression of zonula occludens 1. (a) The cell elongation index was determined by image analysis. The typical appearance of cells is shown below the chart. Dataset was analyzed using ANOVA with the Tukey post hoc test (*p* 0.05). All differences were statistically significant. (b) Immunocytochemical detection of zonula occludens 1 (green) and nuclei (blue). Representative images out of two independent biological replicates. The flow direction is from left to right for images from microfluidic chips.

Based on biocompatibility assays with surface-modified PDMS discs (see above, Figure 3b,c), we have selected plasma treatment and collagen IV coating for further work on the microfluidic chip. For sake of clarity, we tested other surface modifications in microfluidic format as well (Figure S4, see Figure 3c for comparison). Microfluidic chips were seeded with endothelial cells, and the cultivation was done under flow (shear stress of 0.03 Pa). The vital staining of cells grown in channels justify the choice of plasma treatment followed with collagen IV coating in terms of excellent viability and uniform coverage with cells (Figure S4). When a confluent monolayer (see Figure 7) of cells was reached (5 days), post-cultivation image analyses were performed. The cells showed elongation, preferential orientation along with the flow (Figure 8a), and high viability (>90%, similar for static culture and the IBIDI chip) as determined by vital staining with fluorescein diacetate and propidium iodide staining (see Figure S5 in the Supporting Information). The elongation of cells was analyzed quantitatively and compared with cultures grown under static conditions and in a commercial microfluidic chip. The static cell cultures showed the elongation index (a major to minor axis ratio of the circumscribed ellipse) of 2.0 ± 0.01. This parameter was significantly higher (2.9 ± 0.01) for cells growing in an IBIDI microfluidic chip, which served as a reference microfluidic chip. The cells were even more elongated in our microfluidic chips with the cylindrical channels, as the elongation index reached 3.6 ± 0.2 (Figure 8a). Further, the expression of ZO1 was determined by immunocytochemical staining. In the static cell culture, ZO1 was rather diffuse in the cytoplasm. Cells growing in the IBIDI microfluidic chip as well as in our microfluidic chips with the cylindrical channels showed preferential expression of ZO1 along cell borders (Figure 8b).

## Discussion

The most important achievement of our work is the improved uniformity of plasma-based surface modification of closed microfluidic channels within a PDMS chip. Such surface modification is an integral part of a more complex procedure, which allows for rapid prototyping of microfluidic chips and promotes a stable culture of endothelial cells under flow.

The possibility of rapid prototyping was ensured by using 3D printing to prepare a mold for PDMS casting. Since the resolution of the used FDM technique is limited in terms of the production of smooth surfaces, the 3D-printed ABS mold was polished by acetone vapor etching.^35^ On the contrary, the etching of precise alignment structures was prevented with water-soluble PEG masking. Such surface modification secured both intact alignment structures and resulted in a very smooth and optically clear bottom surface of the PDMS cast. Hence, the bonding of the PDMS cast to the glass support was enabled. The molding elements were coated with BSA to secure safe removal from the PDMS cast without major mechanical damage to the luminal surface of the channels. This relates to the covalent bonding of a protein to the PDMS surface during the curing process.^36^ Thus, it prevented tight contact of PDMS with the surface of the molding elements.

The channel diameter (400 μm) reflected the size of the molding element well. Irregularities of the diameter were less than 5 μm along a single channel. Electron microscopy imaging revealed submicrometer scratches, which were probably a result of pin removal. Such precision is better than the competing approach based on the deposition of PDMS into the rectangular channel, which resulted in irregularities up to ±10 μm as deducted from the data in Fiddes et al.^20^ Other major competing approaches rely on removable cylindrical molding elements which are dissolved,^22^ melted,^37^ or removed^10,24^ after PDMS curing. The drawback is the imprecision of the dissolvable element or its long dissolution time and possible damage due to PDMS swelling when using a low-polarity organic solvent.^1^ The melting of the molding element resulted in damage to the PDMS surface as well.^37^ A similar approach to the one presented in this paper utilized removable molding elements such as tungsten wire^10^ or optical fibers.^23^ We have substituted such material with more readily available stainless steel entomological pins (250 to 700 μm, in 50 μm increments) and standard gauge hypodermic needles (range 184–4572 μm).

The chosen material, PDMS, is excellent in transparency and has very high chemical stability under physiological conditions (pH ∼ 7.4, presence of salts, 37 °C). It is, however, highly hydrophobic.^1^ To overcome this cell adhesion restricting property, several surface modifications were tested using PDMS discs before application to the closed channel in the PDMS chip. Contrary to literature data, silanization with TEOS to form a thin hydrophilic silica layer^6^ showed a minimal effect if applied directly to PDMS or after its liquid-mediated surface oxidation. The high-frequency low-pressure air plasma-mediated surface oxidation (further referred to as plasma treatment)^38^ was more effective. The plasma was created using a portable electromassage device similar to the work of Haubert^39^ to keep the procedure simple. However, the discharge was applied directly into the channel enclosed within a PDMS chip. The procedure of the plasma treatment of the luminal surface of the channels within the microfluidic chip was inspired by the work of Dixon and Takayama^2^ but without a need for special gas (helium).^4^ Hence, it was possible to reduce costs and promote the availability of the approach. The plasma treatment of the luminal surface resulted in an even lower water contact angle as compared to the flat surface of the PDMS disc. This is due to the confinement of the water meniscus into a narrow space.

Moreover, the issue of non-uniform plasma surface modification reported previously^2^ was addressed using a high-frequency discharge at low pressure.^27^ An assay with protein adsorption showed minor irregularities within ±10%. The resulting relatively even surface modification is a major improvement compared to a previous report on plasma-based luminal surface modification.^2^ In our case, the plasma treatment did not produce major changes in the channels’ luminal surface topography contrary to previous reports.^2−4^ The collagen coating produced an additional layer on the surface, as demonstrated previously.^12^ Such a layer is capable of masking minor irregularities left by plasma treatment. The reversion of the PDMS surface to a hydrophobic one was prevented through its contact with the polar hydrophilic environment,^1^ like ethanol and/or distilled water. In later stages, the reversion was hindered by contact with the collagen IV coating and cell culture media.

To make the surface of the material biocompatible, it needs to be made hydrophilic and further functionalized by the deposition of proteins that can be recognized by cell adhesion receptors.^9^ Collagens are commonly present in the basal lamina, which supports the adhesion of endothelial cells in vivo. Further, they are able to form 3D networks, which makes them good candidates for protein coating to support cell adhesion.^9^ Surprisingly, the endothelial cells growing on collagen IV coated native PDMS discs showed the highest cell culture viability (intracellular ATP assay, Figure 3b), which was even slightly higher than in the case of plasma-treated PDMS with collagen IV coating. Since the cell viability (vital staining, Figure 3c) showed no difference between these variants, the abovementioned discrepancy could be attributed to differences in growth dynamics. Further, the long-term cell culture stability was limited on native PDMS coated with collagen IV. This was improved when the PDMS surface was made hydrophilic by plasma treatment right before collagen IV coating (Figure 3b). The plasma treatment before collagen IV coating reduced the amount of collagen IV bound to the PDMS surface. It could be explained by a thinner collagen layer formed on a hydrophilic surface.^12^ The collagen IV, like many other proteins, takes a native conformation on the hydrophilic surface. Under such conditions, it provides better support to endothelial cell adhesion since it creates a uniform non-fibrillary network.^12^ In other studies, PDMS microfluidic devices for cell cultures were coated with various other proteins, for example, fibronectin,^10^ matrigel,^11^ or gelatine,^13^ but our previous study with fibroblasts and cardiomyocytes indicated collagen IV to be a versatile coating material.^32^

To prepare a bio-mimicking model of a blood vessel suitable to study aspects of cardiovascular diseases, the whole luminal surface of a channel has to be covered with a confluent monolayer of endothelial cells.^16^ The channel diameter of 400 μm was chosen to enable direct cell seeding and subsequent perfusion.^40^ To obtain a uniform cell coverage, we used a rotation of the chip during the seeding procedure, similar to other published approaches, e.g.,^11,20,23^ the cells were cultivated under constant flow until the monolayer was obtained. Based on vital staining we proved that the cells within the channels were highly viable (see Figures S4 and S5). Moreover, the cells also showed physiological response to flow since they were elongated and oriented along with the flow.^10,16^ The response was very similar as in the reference microfluidic chip. Further, the cells in our microfluidic chip expressed ZO1 protein along cell border. Such localization of ZO1 is a marker of correct attachment of cytoskeleton to tight junctions between cells and indicates proper response to flow in endothelial cells.^41^ The response was very similar to cells in the reference IBIDI chip (Figure 8b).

## Conclusions

We have introduced the high-frequency low-pressure air plasma surface modification of seamless channels enclosed within a PDMS microfluidic chip in order to promote a stable culture of endothelial cells under flow. The plasma treatment modified the surface more uniformly than in the previous work. The preparation of the chip combined 3D printing with commonly available materials. Such a setup enables a higher degree of design freedom and a possibility to rapidly prototype. Plasma treatment in combination with a collagen IV coating created a biomimetic surface for efficient adhesion of endothelial cells, which were highly viable and showed physiological response to flow.

## Supporting Information Available

*P*-values for surface modification comparison and for viability assays; comparison of endothelial cell viability inside the channel in our microfluidic chips, IBIDI chip, and static cell culture (PDF)
3D structures of a mold used in our work (ZIP)
