# Supplementary material for: Luminal Surface Plasma Treatment of Closed Cylindrical Microchannels: A Tool toward the Creation of On-Chip Vascular Endothelium
Source: ACS Biomater Sci Eng. 2023 Apr 27;9(5):2755–63. doi: 10.1021/acsbiomaterials.2c00887 (PMC10170472; doi:10.1021/acsbiomaterials.2c00887)
Supplement: Supplementary file 1 — ab2c00887_si_001.pdf [file ab2c00887_si_001.pdf]

Supporting information:

Luminal surface plasma treatment of closed cylindrical microchannel: A tool towards the creation of on-chip vascular endothelium

Marek Černík, Kamila Poláková, Lukáš Kubala, Andrea Vítečková Wünschová, Anna Mac Gillavry Danylevska, Michaela Pešková, Jan Víteček

Number of pages: 7

Number of tables: 3

Number of figures: 5

Table S1 P-values for wetting angle (figure 3a). Statistically significant values are in red.

	PDMS	Piranha	TEOS	Piranha+TEOS	Plasma
PDMS		0.000129	0.000595	0.000129	0.000129
Piranha	0.000129		0.014569	0.751362	0.000129
TEOS	0.000595	0.014569		0.000456	0.000129
Piranha+TEOS	0.000129	0.751362	0.000456		0.000129
Plasma	0.000129	0.000129	0.000129	0.000129	

Table S2 P-values for ATP test (figure 3b). Statistically significant values are in red.

	control	PDMS	PDMS + col I	PDMS + col IV	plasma	plasma + col I	plasma + col IV
control		0.003759	0.087351	0.360029	0.677552	0.258959	1.000000
PDMS	0.003759		0.590462	0.000241	0.067383	0.253242	0.003866
PDMS + col I	0.087351	0.590462		0.001839	0.749302	0.992092	0.089809
PDMS + col IV	0.360029	0.000241	0.001839		0.025102	0.005840	0.352397
plasma	0.677552	0.067383	0.749302	0.025102		0.980017	0.686792
plasma + col I	0.258959	0.253242	0.992092	0.005840	0.980017		0.265132
plasma + col IV	1.000000	0.003866	0.089809	0.352397	0.686792	0.265132	

Table S3 P-values for FDA/PI staining (figure 3c). Statistically significant values are in red

.	control	PDMS	PDMS + col I	PDMS + col IV	plasma	plasma + col I	plasma + col IV
control		0.000137	0.015133	1.000000	0.999944	0.999999	1.000000
PDMS	0.000137		0.170892	0.000136	0.000147	0.000141	0.000137
PDMS + col I	0.015133	0.170892		0.014089	0.031721	0.021747	0.016081
PDMS + col IV	1.000000	0.000136	0.014089		0.999905	0.999998	1.000000
plasma	0.999944	0.000147	0.031721	0.999905		0.999999	0.999966
plasma + col I	0.999999	0.000141	0.021747	0.999998	0.999999		1.000000
plasma + col IV	1.000000	0.000137	0.016081	1.000000	0.999966	1.000000	

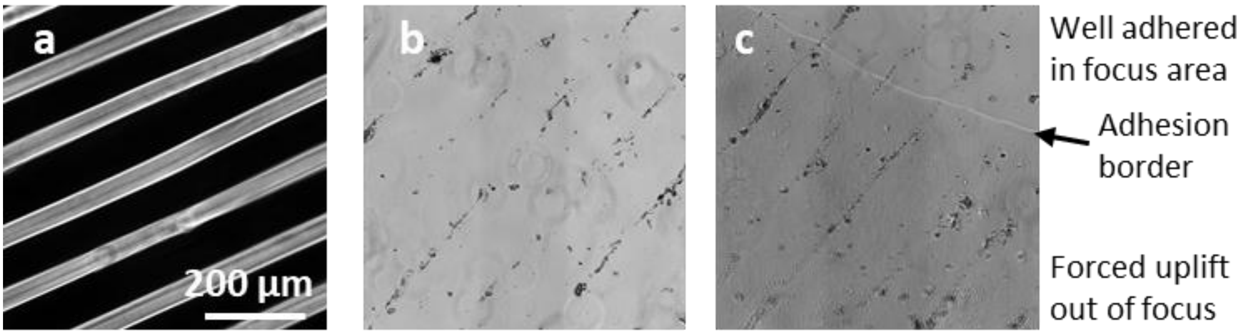

Figure S1 Demonstration of adhesion of PDMS cast on a glass slide. PDMS was cast into 3D printed mold (nozzle size 250 µm, resolution 150 µm) without (a) or with (b,c) acetone vapor polishing. The contact of the cast with a glass slide was evaluated using an optical microscope in phase contrast mode. There were major traces imprinted to the surface of the PDMS cast and there was very limited adhesion in case of cast out of unpolished mold (a). However, the cast out of the acetone-polished mold showed tiny traces only and formed good adhesion (b). To support the correct observation of the adhesion formation a forced manual uplift of the PDMS cast was applied (c): the uplifted part went out of the focus and lost the adhesion to surface but the remaining area was still adhered. A clear adhesion border was formed (c).

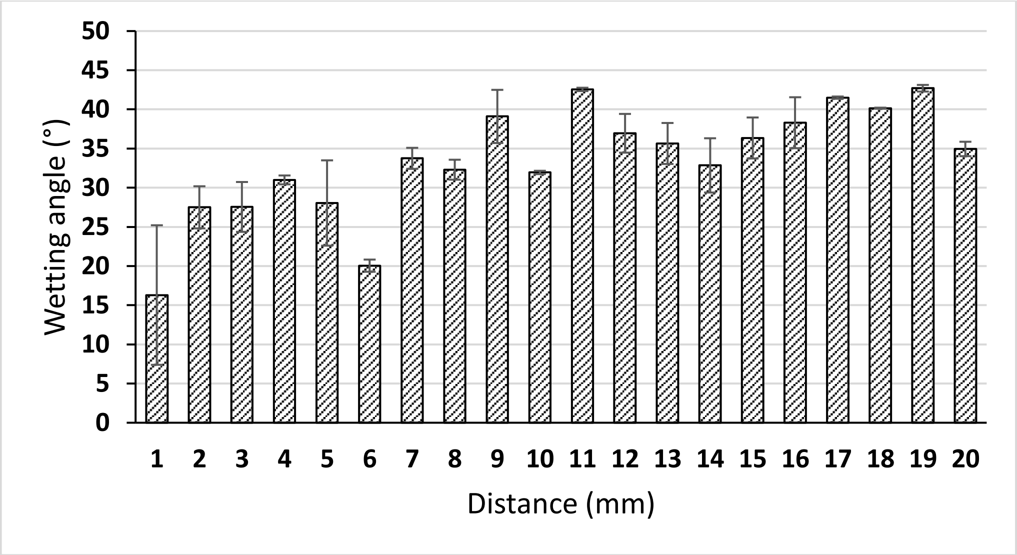

Figure S2 Water wetting angle inside the plasma treated channel. Wetting angle was sampled at 1 mm channel sections. The Error bar indicates standard deviation of the wetting

angles. The approach was charged with high imprecision due to complicated setting of the contact point of the interphase (water - air) at the channel wall (as seen in current figure 4, right side of the channel after 60s of plasma treatment). This was due to inherent light scattering at the side of the circular cross section channel.

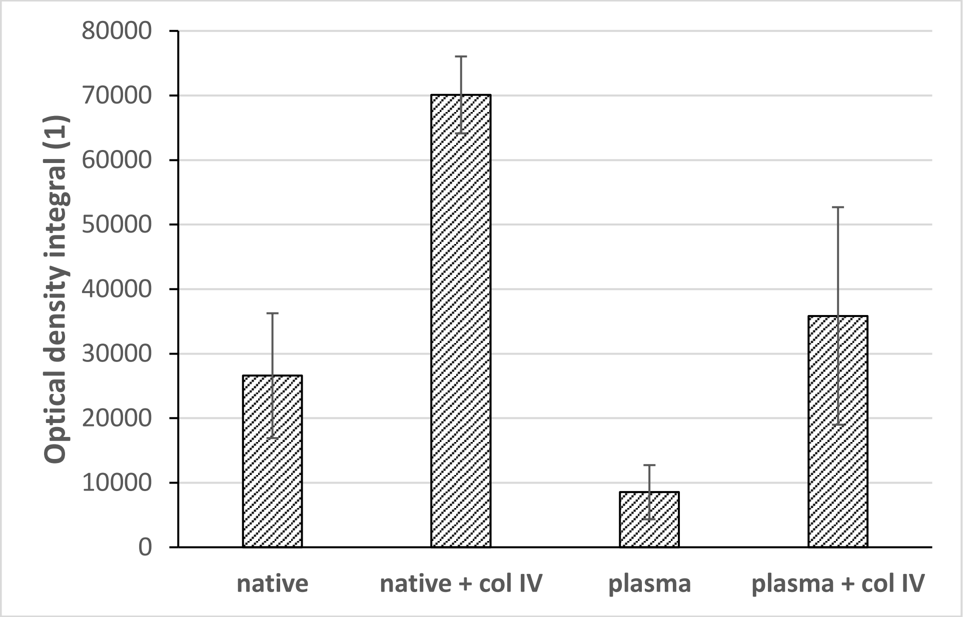

Figure S3: Staining of channels' luminal surfaces for adsorbed collagen IV. Native PDMS as well as plasma treated PDMS was left as it is or coated with collagen IV and stained with Coomassie blue. The staining was evaluated by means of image analysis. Data are displayed as mean value ± standard deviation (N= 6 to 8). Despite the higher background in case of native PDMS the result indicates about 1.5 times higher amount of collagen bound to native PDMS.

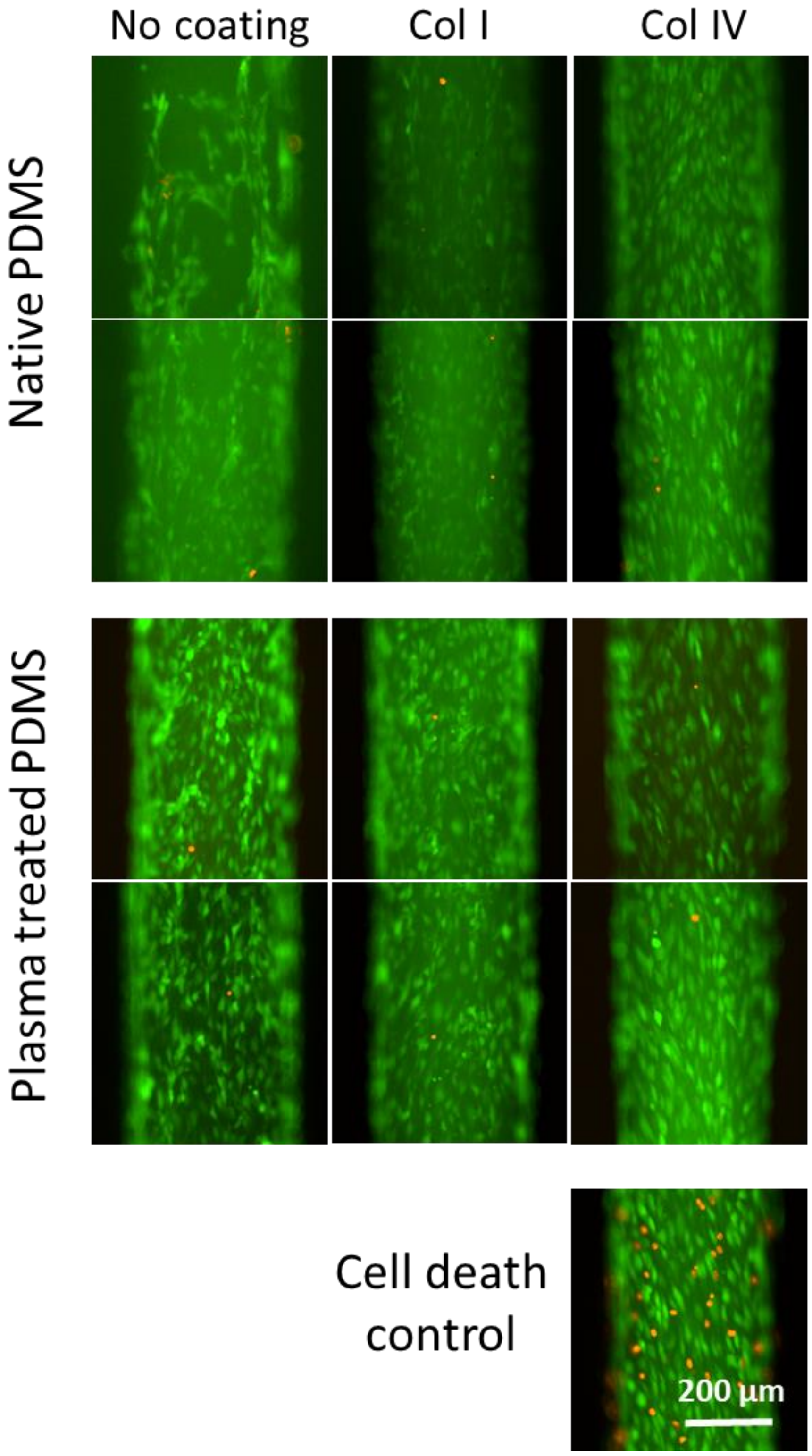

Figure S4 Vital staining of cells in differently coated channels observed with fluorescence microscope, overlay of red (dead cells, propidium iodide), and green fluorescence (living cells, fluorescein). The cells were grown in channels under flow for 5 days. The bottom of the channel was in focus. Additionally, the mechanically damaged cells (by

passing air bubbles through the channel) imaging is provided as a control for the staining procedure of dead cells. Note that a) collagen IV coated variants showed excellent viability and uniform coverage with cells and b) absence of high number of dead cells on native PDMS and native PDMS with collagen coating. The latter mentioned could be attributed to rapid dead cells wash-off since they are poorly attached.

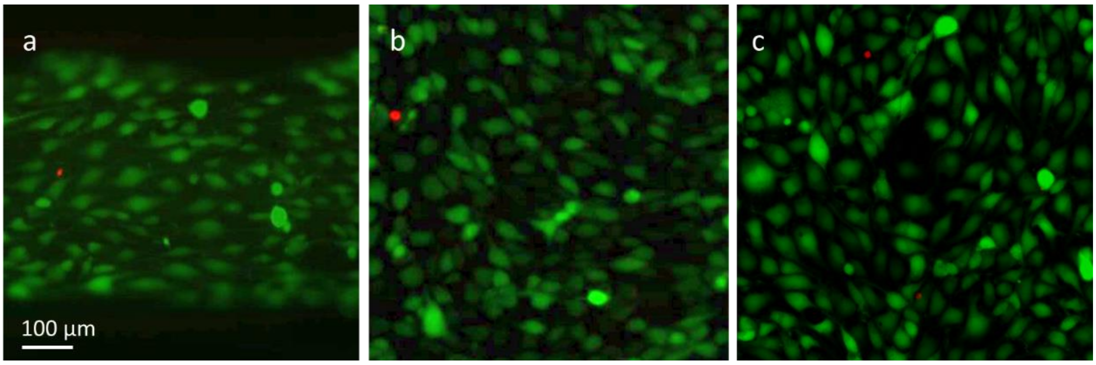

Figure S5 Vital staining of cells using fluorescence microscopy, overlay of red (dead cells, propidium iodide), and green fluorescence (living cells, fluorescein). Cells showed viability over 90%. (a) cells inside the channel of our microfluidic chip, (b) cells inside the IBIDI chip, (c) cells on a cultivation plastic dish.

Supplementary method: Collagen IV staining adsorbed to luminal surface of channels

Four out of total eight channels in the chip were treated with plasma (details in Plasma modification of luminal chip surfaces in Methods of the main text). Two plasma treated and two untreated channels were coated with collagen IV (see Contact angle measurement in methods of the main text). After the coating all of the channels were thoroughly rinsed with phosphate buffered saline (pH 7.4) and dried. Adsorbed protein was stained with 0.1% Coomassie blue R250 solution in 40 % methanol and 10 % acetic acid in water overnight. The

next day the channels were destained with 40 % methanol and 10 % acetic acid in water for 10 min at room temperature. Destaining solution was completely removed from channels and the channels were dried with flow of air. Channels were filled with 47% glycerine in water to match the optical density of PDMS. An image of the chip was taken using the gel documentation device (Alliance 9.7, Uvitec Cambridge). The integral of optical density compared to background was evaluated using ImageJ.
